# Lentiviral Mediating Genetic Engineered Mesenchymal Stem Cells for Releasing IL-27 as a Gene Therapy Approach for Autoimmune Diseases

**Published:** 2014-10-04

**Authors:** Shohreh Hajizadeh-Sikaroodi, Ahmad Hosseini, Ali Fallah, Hajar Estiri, Zahra Noormohammadi, Mohammad Salehi, Sayyed Mohammad Hossein Ghaderian, Haleh Akhavan Niaki, Masoud Soleimani, Bahram Kazemi

**Affiliations:** 1 Science and Research Branch, Islamic Azad University, Tehran, Iran; 2 Cellular and Molecular Biology Research Center, Shahid Beheshti University of Medical Sciences, Tehran, Iran; 3 Mehr Infertility Research Center, Rasht, Iran; 4 Department of Cell Biology and Anatomical Science, Faculty of Medicine, Shahid Beheshti University of Medical Sciences, Tehran, Iran; 5 Systems and Synthetic Biology Group, Mede Bioeconomy Company, Tehran, Iran; 6 Department of Molecular Biology and Genetic Engineering, Stem Cell Technology Research Center, Tehran, Iran; 7 Department of Biology, Faculty of Basic Sciences, Science and Research Branch, Islamic Azad University, Tehran, Iran; 8 Department of Medical Genetics, Faculty of Medicine, Shahid Beheshti University of Medical Sciences and Health Services, Tehran, Iran; 9 Cellular and Molecular Biology Research Center, Babol University of Medical Sciences, Babol, Iran; 10 Department of Hematology, Faculty of Medical Sciences, Tarbiat Modares University, Tehran, Iran; 11 Department of Biotechnology, Faculty of Medicine, Shahid Beheshti University of Medical Sciences, Tehran, Iran

**Keywords:** Autoimmune Disease, Gene Therapy, IL-27, Mesenchymal Stem Cells

## Abstract

**Objective:**

Autoimmune diseases precede a complex dysregulation of the immune system. T helper17 (Th17) and interleukin (IL)-17 have central roles in initiation of inflammation and subsequent autoimmune diseases. IL-27 significantly controls autoimmune diseases by Th17 and IL-17 suppression. In the present study we have created genetic engineered mesenchymal stem cells (MSCs) that mediate with lentiviral vectors to release IL-27 as an adequate vehicle for ex vivo gene therapy in the reduction of inflammation and autoimmune diseases.

**Materials and Methods:**

In this experimental study, we isolated adipose-derived MSCs (AD-MSCs) from lipoaspirate and subsequently characterized them by differentiation. Two subunits of IL-27 (p28 and EBI3) were cloned in a pCDH-513B-1 lentiviral vector. Expressions of p28 and EBI3 (Epstein-Barr virus induced gene 3) were determined by real time polymerase chain reaction (PCR). MSCs were transduced by a pCDH-CMV-p28-IRES- EBI3-EF-copGFP-Pur lentiviral vector and the bioassay of IL-27 was evaluated by IL-10 expression.

**Results:**

Cell differentiation confirmed true isolation of MSCs from lipoaspirate. Restriction enzyme digestion and sequencing verified successful cloning of both p28 and EBI3 in the pCDH-513B-1 lentiviral vector. Real time PCR showed high expressions level of IL-27 and IL-10 as well as accurate activity of IL-27.

**Conclusion:**

The results showed transduction of functional IL-27 to AD-MSCs by means of a lentiviral vector. The lentiviral vector did not impact MSC characteristics.

## Introduction

In recent years stem cell therapy has become a primary aspect of numerous research and clinical projects (1). Stem cell types such as pluripotent (ES, iPS), fetal and adult stem cells are most commonly used as treatments, however despite the advantages, in numerous diseases it is necessary to make genetic alterations to these cells by over expressing or knocking down genes (2). Mesenchymal stem cells (MSCs) with their basic properties can be a good source for cell therapy. In addition these cells have unique features for moderating cell attack and immune system reactions (3). Adipose-derived MSCs (AD-MSCs) are the best source for MSCs that can be used for cell therapy (4). AD-MSCs can be easily isolated from lipoaspirate and possess a stable karyotype as well as high capability for self-renewal in comparison to other sources of MSCs (5). Stem cells in adipose tissue usually comprise up to 3% of the entire cell population, which is 2500 fold more than the frequency of stem cells in bone marrow (6).

Autoimmune diseases are multi-factorial disorders with complicated immune system dysregulation mediated by immune cytokines and immune cells (7). In many autoimmune diseases transforming growth factor beta (TGF-B) and interleukin (IL)-6 induce T helper17 (Th-17) causing IL-23 and IL-17 secretion (8). IL-23 and IL-17 can persuade special CD4+ with CCR2+ and CCR5-effector T cells which have been identified as major agents for inducing autoimmune disease in a mouse model (9). As mentioned, down-regulation of Th17 or IL-17 can be an effective therapy for treatment of many autoimmune diseases (10). Previous studies have confirmed that IL-27 is a strong suppressor of Th17 and IL-17. Therefore overexpression of IL-27 may be a good optional th erapy against autoimmune diseases (11).

There are many autoimmune diseases which all have the same mechanism of pathogenicity, thus one approach can be used as a general treatment for these diseases (12). In the present study we report a construct that can be used for a gene therapy approach based on the suppression of IL-17 by IL- 27 producer cells.

## Materials and Methods

### IL-27 construct in the lentiviral vector

In this experimental study, we purchased two subunits of mouse IL-27 (p28 and EBI3) cDNA from Open Biosystems (Huntsville, AL, United States). Both genes were cloned in pCDH- 513B-1 (System Bioscience, Mountain View, CA, United States) combined with an internal ribosome entry site (IRES) sequence arranged as p28-IRES-EBI3 under a cytomegalovirus (CMV) promoter. pCDH-513B-1 have copGFP (copepod green fluorescent protein) for fluorescent tagging and puromycin for selecting stably transduced cells. All cloning procedures were performed according to the common digestion-ligation protocol. Polymerase chain reaction (PCR) was carried out for three fragments with Xba1-p28-Nhe1, Nhe1-IRESBamH1 and BamH1-EBI3-Not1 overhanging. Then, all fragments were separately cloned in pCDH-513B-1. We verified the pCDH-CMVp28- IRES-EBI3-EF1-copGFP-Pur construct by digestion and subsequent sequencing.

### Recombinant lentiviral production

Recombinant lentivirus was produced according to the TRONOLAB protocol with some modifications (13). Briefly, 1×10^6^ HEK-293T cells (Invitrogen, Carlsbad, CA, United States) were cultured in a 10 cm plate in Dulbecco’s Modified Eagle’s Medium (DMEM) (GIBCOBRL, Tokyo, Japan) with 10% Fetal bovine serum (FBS) (GIBCO-BRL, Tokyo, Japan) one day prior to transfection. We replaced the medium 2 hours before transfection with fresh medium. Ca_3_(PO_4_)_2_ buffer that contained 21 μg of pCDH-CMV-p28-IRES-EBI3-EF1-copGFPPur, 21 μg of pCMV-dR8.2, 10.5 μg of pMD2, 33 μl of TE 1X, 105 μl of 2.5 M CaCl_2_, and 1050 μl of 2x Hank’s buffered salt solution (HBSS) was used for one 10 cm plate. We added the 2X HBSS during the time the solution was vortexed. Transfection medium was replaced with fresh medium within 14 hours after transfection. Medium with viruses was collected after 24, 48 and 72 hours and centrifuged at 15000 rpm, then filtered through a 0.45 μm filter before transduction.

### Mesenchymal stem cell (MSCs) isolation from human adipose tissue, culture and differentiation

Adipose tissue was obtained from lipoaspirate plastic surgery performed at clinics according to a Bioethics Agreement of the Shahid Beheshti University of Medical Science and Stem Cell Research Center Committee. Adipose tissue was washed three times with phosphate buffered saline (PBS) that contained 3X penicillin/streptomycin and amphotericin until a clear tissue was attained. DMEM medium that contained dispase (50 U/ml)-Collagenase I (250 U/ml; Sigma-Aldrich, St. Louis, MO) were added to the adipose tissue, after which the solution was shaken for 30 minutes at 37˚C. The solution was centrifuged at 1500 rpm and the supernatant was discarded. The plated cells were kept. RBC was lysed by erythrocyte lysis buffer for 5 minutes at 37˚C, and then centrifuged at 1200 rpm for 5 minutes. The plated cells were suspended in DMEM and distributed in flasks with DMEM that contained 10% FBS for 3 days. For adipogenic differentiation, cells were cultured in DMEM that contained 10% FBS, 0.5 mM isobutylmethylxanthine (IBMX), dexamethasone (10-7 M), insulin (66 nM), and indomethacin (0.2 mM). For osteocyte differentiation the cells were cultured in DMEM that contained 10% FBS, dexamethasone (10-7M), β-glycerol-phosphate (10 mM), and ascorbic acid 2-phosphate (50 μg/ml).

### Adipose-derived mesenchymal stem cells (ADMSCs) transduction by lentivirus

Second passage AD-MSCs were cultured in sixwell cell culture plates, and then washed with PBS before adding fresh recombinant virus. In order to remove all FBS proteins to enable better transduction we used the spinfection method at 2000 rpm for 60 minutes at a temperature of 25˚C. After centrifuging, the plate was placed in a 37˚C incubator; the medium was changed 14-20 hours after spinfection.

### Expression of IL-27 and self-renewing assay with Oct-4

Total RNA extraction and cDNA synthesis from 2×10^6^ AD-MSCs and lentiviral engineered ADMSCs were carried out by Qiagen (Alameda, CA, United States), RNA extraction and cDNA kits (Waltham, MA, United States), respectively, according to the manufacturers’ protocols. cDNA was used for quantitative real time PCR. Expressions of octamer-binding transcription factor 4 (Oct-4), IL-27 and EBI3 were evaluated in ADMSCs and lentiviral engineered AD-MSCs. TATA- binding protein (TBP) expression was considered to be the endogenous reference gene. Primer sequences used this studied are provided in table 1.

**Table1:** Primer sequences used for quantitative real time PCR


Primer	Forward	Reverse

**IL-27**	5' AGACTCTGCTTCCTCGCTA 3'	5' CCTCCTCCTTTGAACATTT 3'
**EBI-3**	5' TGAGCGAATCATCAAGCC 3'	5' GTTTCCCATAATCTGTGAGG 3'
**Oct-4**	5' CGGCGTATGAGTTGTGTG 3'	5' GGTGATCCTCTTCTGCTTC 3'
**TBP**	5'CTCTCTGCTCCTGTTCG 3'	5'ACGACCAAATCCGTTGACTC 3'


### Bioassay of IL-27

Secretion and function of IL-27 were examined by bioassay using naive T cells from a C57BL/6 mouse that was co-cultured with COS-7 cells transduced with recombinant virus derived from pCDHCMV- p28-IRES-EBI3-EF1-copGFP-Pur and selected for puromycin at a concentration of 2 μg/cc. The C57BL/6 mouse was killed according to the laboratory animal protocol. The spleen was removed and cut in small pieces then digested with dispasecollagenase (100U/ml) for 10 minutes and passed through 0.45 μM filter. Cells were collected by centrifugation at 1200 rpm and RBCs were lysed by a RBC lysis buffer. After three days, naive T cells were cultured in fresh RPMI 1640 medium and co-cultured with COS-7 that was engineered by a recombinant virus (COS7/IL-27). Total RNA was extracted from T cells co-cultured with COS-7 and COS-7/ IL-27. Expression of IL-10 was appraised as a downstream gene in the IL-27 signal transduction pathway by real time PCR. Glyceraldehyde-3-phosphate dehydrogenase (GAPDH) expression was used as an endogenous reference gene. The following primers were used for IL-10 and GAPDH: IL-10 forward: 5´AATAAGAGCAAGCCAGTG3´ and reverse: 5´CCAGCAGACTCAATACAC3'; and GAPDH forward: 5'CCACAACTCTTCCATTCTC3' and reverse: 5'CCAAGATTCACGGTAGATAC 3'.

### Ethical considerations

Human adipose tissue was obtained following informed consent in accordance with the Declarations of the Shahid Beheshti University of Medical Science and Stem Cell Research Center Committee.

## Results

### IL-27 lentiviral construct and recombinant viral particle production

We cloned mouse p28 and EBI3 cDNA in a pCDH-513B-1 lentiviral vector. Digestion with XbaI-NotI showed that cloning was successful (Fig l). The construct was co-transfected with the helper packaging vector mediated Ca_3_(PO_4_)_2_ protocol. The transfect efficiency was more than 90% (Fig 2). The viral particle titer was approximately 1.5-2×10^6^.

**Fig 1:**
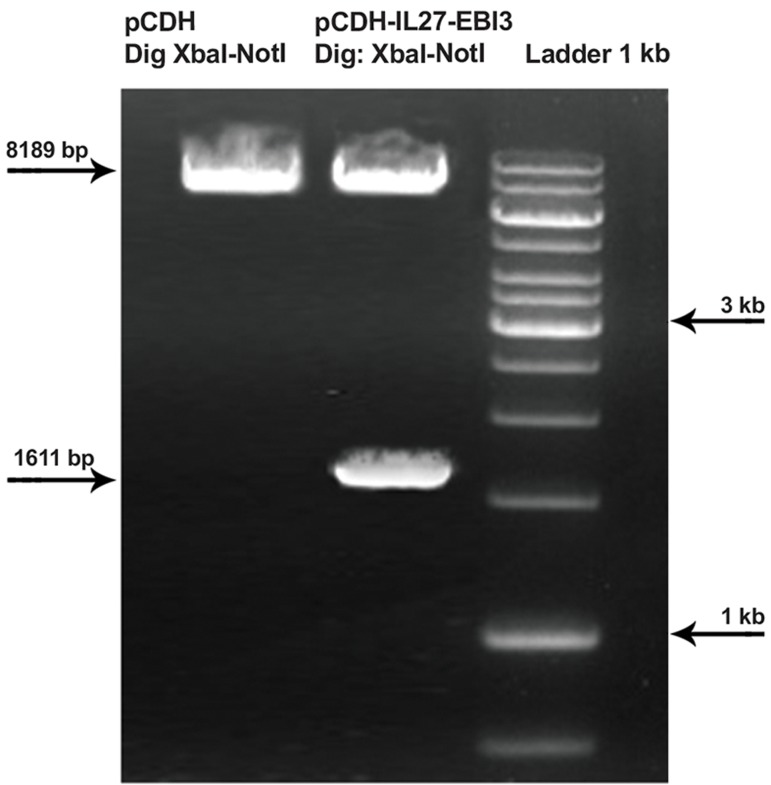
p28 and EBI3 genes inserted into the pCDH-513B-1 lentiviral vector. Digestion with XbaI-NotI showed an 8189 bp length of the pCDH-513B-1 backbone. The presence of a 1611 bp segment related to p28-IRES-EBI3and a segment of an 8189 bp related to pCDH-513B-1 confirmed that the cloning was established. A genetic map confirmed these data.

**Fig 2:**
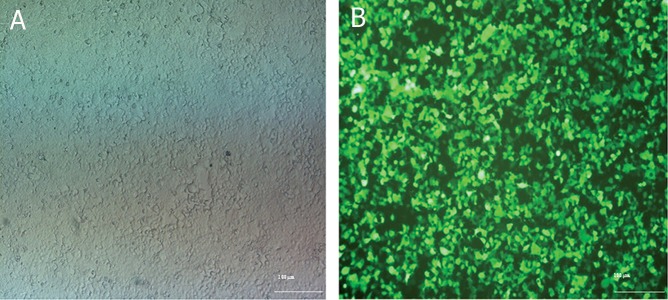
Transfection of HEK-293T for achieving viral particles. Panel A shows the HEK-293T culture. Panel B represents HEK- 293T at 18 hours after transfection by pCDH-CMV-p28-IRES-EBI3-EF1-copGFP-Pur. High expression of GFP in HEK-293T shows the high rate of transfection.

### Adipocyte stem cell isolation, transduction of adipose-derived mesenchymal stem cells (ADMSCs) with recombinant lentiviral particles

**Fig 3:**
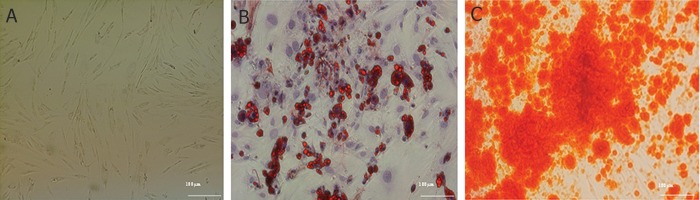
Isolation of mesenchymal stem cells (MSCs) from adipose tissue and characterized by differentiation. Panel A shows passage 2 adipose-derived MSCs (AD-MSCs), Panel B represents oil red staining of passage 2 AD-MSCs that differentiated into adipocytes. The vesicle that contained oil is visible in cells which showed adipogenic differentiation and Panel C shows the passage 2 AD-MSCs that were cultured in osteogenic differentiation medium and stained with alizarin red. Alizarin red stained the calcium deposits which confirmed osteogenic differentiation.

**Fig 4:**
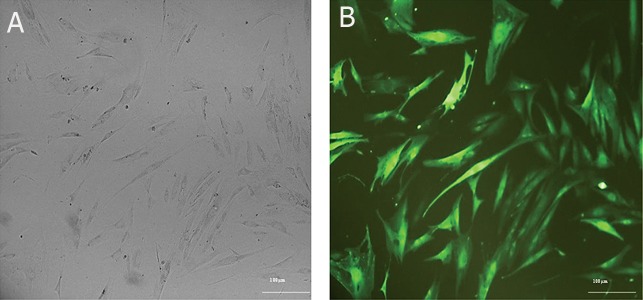
Transduction of adipocyte-derived mesenchymal stem cells (AD-MSCs) by lentiviral particles. Panel A shows AD-MSCs prior to transduction and Panel B shows transduced AD-MSCs by pCDH-CMV-p28-IRES-EBI3-EF1-copGFP-Pur lentiviral vector. The numerous green cells and GFP expression indicate a high level of transduction.

Adipocyte cells were isolated from liposuction tissue with mechanical and enzyme digestion. Multipotency of the cells was confirmed by their differentiation into adipocyte and osteocyte cells. Alizarin Red staining confirmed the presence of osteocytes (Fig 3A) and oil red staining showed the adipocyte properties after differentiation (Fig 3B). AD-MSCs showed over 70% efficiency when transduced by lentiviral particles (Fig 4). Transduced AD-MSCs were selected via puromycin. The selection curve determined that 2 μg of puromycin was sufficient to generate approximately 95% pure transduced cells after 3 days. The GFP marker provided a good index for the transfection, transduction and purification processes.

### Gene expression profiles

**Fig 5:**
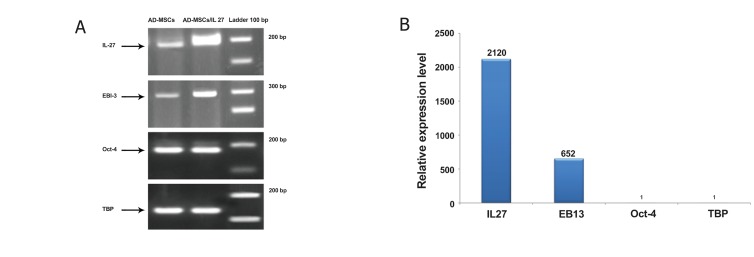
Expressions of IL-27, EBI3, and Oct-4 in adipose-derived mesenchymal stem cells (AD-MSCs) and transduced ADMSCs/ IL-27. Panel A shows the result of real time-PCR that confirmed the presence of a definite transcript by gel resolution. Expressions of p28 and EBI3 increased in transduced AD-MSCs/IL-27 compared with control AD-MSCs. Oct-4 expression did not show any significant difference. TBP was used as the RNA integrity control. Panel B represents the level of p28, EBI3 and Oct-4 expressions in AD-MSCs and AD-MSCs/IL-27 compared by real time PCR. The level of p28 expression increased 2000- fold, whereas EBI3 expression increased 650-fold.

Real time PCR showed expression of p28 increased 2000-fold and EBI3 increased 650-fold in transduced AD-MSCs compared with the control AD-MSCs (Fig 5). IRES sequencing between p28 and EBI3 had a 3-fold decrease in EBI3 expression in reference to p28. Expression of Oct-4 in transduced AD-MSCs confirmed that AD-MSCs preserved their self-renewing potency. Lentiviral transduction did not affect the mesenchymal properties.

### IL-27 functional assay

**Fig 6:**
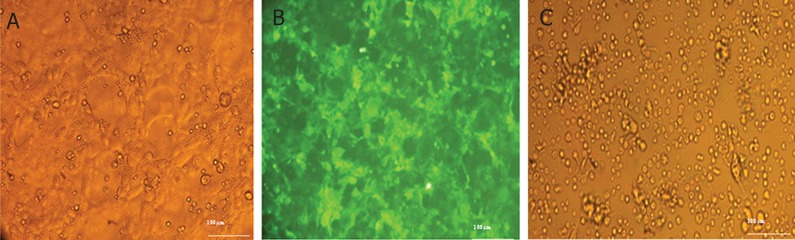
T cell isolation and co-culture with COS-7. Panel A shows COS-7 cells prior to transduction. Panel B shows the transduced COS-7 by pCDH-CMV-p28-IRES-EBI3-EF1-copGFP-Pur lentiviral vector. COS-7 and COS-7/IL-27 were inactivated by mitomycin C. Panel C shows T cells isolated from the spleen of a C57BL/6 mouse.

**Fig 7:**
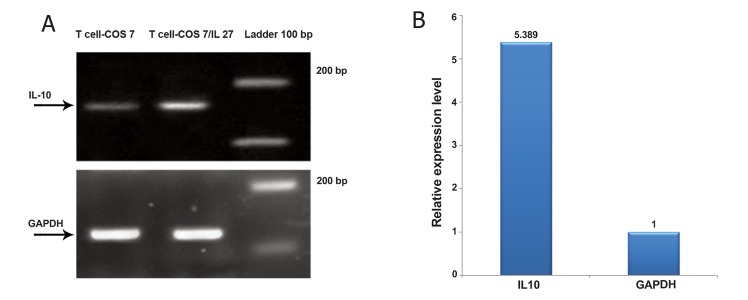
Expression of IL10 as an IL-27 biological activity assay. Panel A shows the results of real time-PCR. IL-10 is overexpressed in T cell-COS7/IL-27 compared with T cell-COS7. GAPDH served as the endogenous reference gene. Panel B shows the level of IL10 expression in T cells cultured with COS-7/IL-27 increased 5-fold compared with COS-7 by real time PCR.

We examined the biological activity of IL-27 that was secreted from COS-7 cells. Naive T cells produced larger amounts of IL-10 when co-cultured with COS-7/IL-27 cells compared to naive T cells co-cultured with COS-7 (Fig 6). Real time PCR showed a 5-fold higher expression of IL-10 in T cells co-cultured with COS-7/IL-27 compared with T cells co-cultured COS-7 (Fig 7).

## Discussion

Autoimmune diseases are complex disorders with an immunological basis that are dependent on cytokines that are good targets for gene therapy (14). A great deal of research has shown that the use of MSCs as a therapy can be possible (15). In this study, we have shown that human MSCs derived from adipose tissue can be considered as a cellular vehicle for ex-vivo gene therapy. We inserted two subunits of IL-27 (p28 and EBI3) into a lentiviral vector that has a bright form of green fluorescent protein (GFP). Numerous studies have shown that copGFP as a new version of GFP with boosted fluorescent is more useful for enhanced in vivo and *in vitro* visualization (16). The findings of this study have shown that high transduction effectiveness was approximately 95% based on the puromycin selection which is beneficial for an ideal therapeutic application.

Real time PCR verified overexpressed IL-27 because of the leniviral CMV promoter. P28 and EBI3 combined with IRES, thus the expression of the EBI3 subunit decreased by one third compared to p28. This result was similar to other published studies related to IRES effectiveness on gene expression (17). p28 is a core subunit of IL-27 and sufficient for anti-inflammation function whereas EBI3 is a transmembrane protein to have efficient secret and purpose of IL-27 (18), therefore low expression level of EBI3 does not make any effect on IL-27 functional activity.

Our results and those of other similar studies (19, 20) did not show any negative effects of lentiviral transduction and transgene expression on AD-MSCs pluripotency properties. Transcription factor Oct-4 expression was similar in both ADMSCs and AD-MSCs/IL-27.

Murugaiyan et al. have shown that human IL-27 induces generation of T cells which secrete large amounts of IL-10 (21). Real time PCR can show expression level of genes however bioassay definitely verifies gene expression effectiveness. There are a small number of reports that have used bioassays for functional activity confirmation (22). The present study has demonstrated overexpression of IL-10 in T cells cultured with COS-7/IL-27 which represented the correct functionality of IL-27.

## Conclusion

IL-27 can be successfully transduced to ADMSCs by means of a lentiviral vector. IL-27 was functional. The lentiviral vector did not affect MSCs characteristics.
